# Early discontinuation of empirical antibiotic treatment in neutropenic patients with acute myeloid leukaemia and high-risk myelodysplastic syndrome

**DOI:** 10.1186/s13756-020-00729-2

**Published:** 2020-05-27

**Authors:** F. A. Niessen, M. S. M. van Mourik, A. H. W. Bruns, R. A. P. Raijmakers, M. C. H. de Groot, T. van der Bruggen

**Affiliations:** 1 Department of Medical Microbiology and Infection Control, University Medical Centre Utrecht, Room G04.614, Heidelberglaan 100, Postbus 85500, 3508 GA Utrecht, The Netherlands; 2 Department of Haematology, University Medical Centre Utrecht, Utrecht, The Netherlands; 3 Department of Clinical Chemistry and Haematology, Division Laboratories, Pharmacy and Biomedical Genetics, University Medical Centre Utrecht, Utrecht, The Netherlands

**Keywords:** Carbapenems, Vancomycin, Febrile neutropenia, Acute myeloid leukaemia, Myelodysplastic syndrome, Antibiotic stewardship, Interrupted time series

## Abstract

**Introduction:**

Current guidelines advocate empirical antibiotic treatment (EAT) in haematological patients with febrile neutropenia. However, the optimal duration of EAT is unknown. In 2011, we have introduced a protocol, promoting discontinuation of carbapenems as EAT after 3 days in most patients and discouraging the standard use of vancomycin. This study assesses the effect of introducing this protocol on carbapenem and vancomycin use in high-risk haematological patients and its safety.

**Methods:**

A retrospective before-after study was performed comparing a cohort from 2007 to 2011 (period I, before restrictive EAT use) with a cohort from 2011 to 2014 (period II, restrictive EAT use). Neutropenic episodes related to chemotherapy or stem cell transplantation (SCT) in patients with acute myeloid leukaemia (AML) or high-risk myelodysplastic syndrome (MDS) were analysed. The primary outcome was the use of carbapenems and vancomycin as EAT during neutropenia, expressed as days of therapy (DOT)/100 neutropenic days and analysed with interrupted time series (ITS). Also the use of other antibiotics was analysed. Safety measurements included 30-day mortality, ICU admittance within 30 days after start of EAT and positive blood cultures with carbapenem-susceptible microorganisms.

**Results:**

Three hundred sixty-two neutropenic episodes with a median duration of 18 days were analysed, involving 201 patients. ITS analysis showed decreased carbapenem use with a step change of − 16.1 DOT/100 neutropenic days (95% CI − 26.77 to − 1.39) and an overall reduction of 21.6% (8.7 DOT/100 neutropenic days). Vancomycin use decreased with a step change of − 13.7 DOT/100 neutropenic days (95% CI − 23.75 to − 3.0) and an overall reduction of 54.7% (14.6 DOT/100 neutropenic days). The use of all antibiotics combined decreased from 155.6 to 138 DOT/100 neutropenic days, a reduction of 11.3%. No deaths directly related to early discontinuation of EAT were identified, also no notable difference in ICU-admission (9/116 in period I, 9/152 in period II) and positive blood cultures (4/116 in period I, 2/152 in period II) was detected.

**Conclusion:**

The introduction of a protocol promoting restrictive use of EAT resulted in reduction of carbapenem and vancomycin use and appears to be safe in AML or high-risk MDS patients with febrile neutropenia during chemotherapy or SCT.

## Introduction

Patients with acute myeloid leukaemia (AML) or high-risk myelodysplastic syndrome (MDS) are treated with intensive chemotherapy and, if indicated, an allogenic stem cell transplantation (SCT). This treatment results in periods of neutropenia with mucositis, making patients vulnerable to severe infections. Due to the mucosal barrier injury, microorganisms from the gastrointestinal tract can translocate to the bloodstream, including Gram-negative bacteria, viridans streptococci and *Candida* spp.. Therefore, patients receive antimicrobial prophylaxis during the period of neutropenia. Despite prophylaxis, more than 50% of these patients become febrile [1, 2].

When a patient develops fever during neutropenia, broad spectrum empirical antibiotic treatment (EAT) is started immediately to rapidly and adequately treat a bacterial infection. Empirical antibiotic options all include coverage of *Pseudomonas aeruginosa*. Commonly used EAT regimes are a carbapenem, piperacillin/tazobactam, ceftazidime or cefepime with or without an aminoglycoside [1, 3].

Current guidelines advocate to continue EAT until the patient has been afebrile for ≥48 h [3] or even as long as the duration of neutropenia [1]. However, fever during neutropenia does not necessarily have a bacterial etiology. Viral and fungal infections are also frequently encountered. In addition, in 30–50% of cases no causative pathogen can be identified [4]. The haematological malignancy, administration of blood products, or mucositis itself might also cause fever [5, 6]. Current protocols advocating continuous EAT in neutropenic fever may therefore lead to overtreatment with broad-spectrum antibiotics and associated risks of side effects and antibiotic resistance. The broad spectrum antibiotics used as EAT are listed by the World Health Organization (WHO) as critically important antimicrobials for human medicine (Watch category of the AWaRe classification) and are considered key targets of stewardship programs and monitoring [7].

Recent studies suggest that protocols with a more restrictive use of EAT can be safely implemented [8, 9]. A prospective study concluded that discontinuation of EAT after 3 days in febrile neutropenia is safe in a subset of hemodynamically stable patients without positive blood cultures. However, no control group was available in this study [10].

As of 2011, a new protocol promoting early discontinuation of EAT adapted from Slobbe et al. [10], had been introduced at the haematology department of the University Medical Centre Utrecht (UMCU). This offered the opportunity to assess with a historical control group the effect of this protocol on carbapenem and vancomycin use and its safety in AML and high-risk MDS patients with febrile neutropenia.

## Methods

### Patient population

This retrospective before-after study was performed with a cohort of haematological patients treated from 2007 to 2014 at the haematology department of the UMCU. The intervention consisted of the introduction of a new local protocol that, in contrast to the previous local protocol, promoted early discontinuation of EAT with carbapenems and discouraged standard empirical treatment with vancomycin. This new protocol was started on January 1st 2011.

All adult patients (≥ 18 years) with the diagnoses AML or high-risk MDS, treated between January 1st 2007 and December 31st2014 in the UMCU with at least one period of prolonged and profound neutropenia were included. Of these patients, only neutropenic episodes related to intensive chemotherapy (including one of the following cytostatic agents: idarubicin, cytarabine, daunorubicin, vincristine, adriamycin, mitoxantrone or etoposide) or allogenic SCT were included. Neutropenic episodes following other chemotherapeutic regimens or unrelated to chemotherapy or allogenic SCT were excluded. Neutropenic episodes between January 1st 2007 and October 1st 2010 (period I) were compared to neutropenic episodes between April 1^st^ 2011 and December 31st 2014 (period II, after starting the new protocol). The period from October 1st2010 to April 1^st^ 2011 was considered a transition period, therefore neutropenic episodes occurring within this interval were excluded. Complete patient data was not available before 2007, therefore the maximal period that could be studied before the intervention was 45 months (taken into account the transition period). A similar length was chosen for study period II, i.e. 45 months, making the total study period 8 years (2007–2014).

### Definition of profound and prolonged neutropenia

Neutropenia was defined as at least two consecutive neutrophil measurements of < 0.5 × 10^9^cells/L within 90 days. A single neutrophil count above 0.5 × 10^9^cells/L was ignored, if flanked by neutrophil counts below < 0.5x10^9^ cells/L within 1 week. Only prolonged neutropenic episodes with a duration of 7 days or more, occurring within 14 days after start of chemotherapy or conditioning for allogenic SCT, were taken into account. These neutropenic episodes are frequently accompanied by mucositis. If chemotherapy or allogenic SCT was started during a period of pre-existing neutropenia, the ensuing neutropenia was considered to be (at least partially) treatment- related if the neutropenia continued for at least 7 days after start of treatment.

### Prophylaxis and antimicrobial treatment; old protocol

**Fig. 1:**
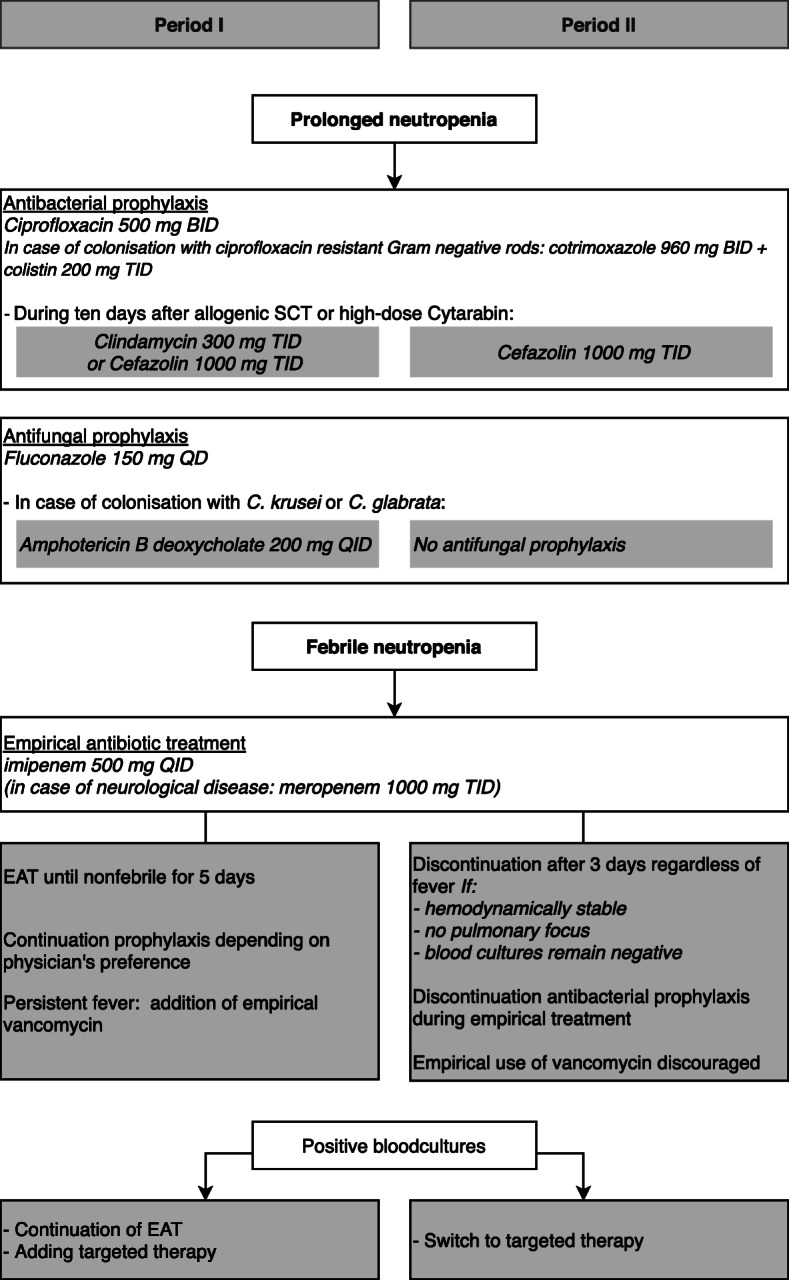
Prophylaxis and antibiotic treatment protocol

Before January 2011, prophylaxis during an episode of prolonged neutropenia consisted of oral ciprofloxacin 500 mg BID and oral fluconazole 150 mg QD (Fig. 1). When patients were colonized with ciprofloxacin resistant Gram-negative bacteria, cotrimoxazole (960 mg BID orally) combined with colistin (200 mg TID orally) was used as prophylaxis. Streptococcal prophylaxis was added when patients received high dose cytarabine (clindamycin 300 mg TID orally) or busulfan/cyclophosphamide (cefazolin 1000 mg TID i.v.). In case of colonisation with *Candida glabrata* or *Candida krusei*, patients received amphotericin B deoxycholate orally (200 mg QID), instead of fluconazole. When a patient developed fever, defined as a single temperature measurement > 38.6 °C or > 38.3 °C for more than an hour, EAT with imipenem 500 mg QID i.v. was administered. Antibiotic prophylaxis was either stopped or continued during EAT depending on the preference of the treating physician. If fever persisted, empirical vancomycin was added according to the physician’s judgment. Imipenem was continued until patients were afebrile for at least 5 days. If blood cultures became positive, EAT was continued for at least 10 days, and depending on the pathogen/clinical suspicion, targeted antibiotics were added (e.g. vancomycin). In case of neurological disease / neurological involvement or symptoms, patients received meropenem (1000 mg TID i.v.) instead of imipenem because of an increased probability of convulsions [11].

### Prophylaxis and antibiotic treatment; new protocol

In January 2011, a protocol on restrictive EAT use was introduced, adapted from the protocol described by Slobbe et al [10]. The new protocol was prepared by a subgroup of haematologists and medical microbiologists and subsequently discussed with the full haematology staff and the hospital antibiotic committee. The final protocol was endorsed by all parties and published on a haematology protocol site. Afterwards, during multidisciplinary meetings held twice weekly, the new protocol and the adherence was frequently brought to attention. Bacterial prophylaxis did not change except that streptococcal prophylaxis consisted of cefazolin only (1000 mg TID i.v.). Fluconazol was given, but in case of colonisation with *C. glabrata* or *C. krusei* no antifungal prophylaxis was administered. In case of febrile neutropenia, imipenem was started and discontinued after 3 days if blood cultures remained negative, irrespective of fever, on condition that the patient was hemodynamically stable and there was no suspicion of pulmonary infection with an (at that moment) unknown etiology (Fig. 1). The use of vancomycin as standard EAT was discouraged, except in case the patient was hemodynamically instable. When blood cultures became positive, therapy was targeted on the pathogen and EAT was discontinued. During EAT, antibacterial prophylaxis was discontinued. If a patient became febrile for a second time during the same neutropenic episode, imipenem was restarted (after collection of blood cultures and repeated physical examination) and stopped as described above. According to the protocol, EAT was not restarted for additional 3rd febrile episodes during the same neutropenic period, provided that the patient remained hemodynamically stable.

### Data sources

A primary database containing patient data extracted from hospital electronic patient record systems was set up using the Research Data Platform in the UMCU. This database consisted of all patients linked to the diagnosis or treatment of AML or MDS. Data of prescribed antibacterial therapy and cell-count data used to identify neutropenic episodes, were derived from the Utrecht Patient Oriented Database (UPOD) [12]. Data of administered cytostatic agents and data concerning allogenic SCT in our patient selection was collected from the in-hospital pharmacy department of the UMCU and the treatment files of the haematology department, respectively. Data of positive blood cultures was derived from the General Laboratory Information Management System (GLIMS). This study was performed in accordance with the ethical standards of our centre.

### Outcome measurements

The primary outcome was carbapenem and vancomycin use within neutropenia following chemotherapy or conditioning for allogenic SCT in AML and high-risk MDS patients. Because neutropenia following chemotherapy or SCT was considered as the period that patients were most at risk for severe infections and the intervention involved a policy change regarding antibiotic use within this period of neutropenia, antibiotic use was expressed as total days of therapy (DOT) per 100 neutropenic days. Interrupted time series (ITS) analysis was performed to assess pre-existing trends, the immediate effect of intervention (step change) and the sustainability of this effect in period II. In addition, the overall use of carbapenems and vancomycin within period I, calculated as the sum of DOT divided by the sum of neutropenic days * 100, was compared to the overall use in period II.

As secondary outcome, the use of other antibiotic agents was analysed, expressed as DOT per 100 neutropenic days. Total antibiotic use consisted of the sum of DOT of all antibiotics combined. If a patient used 2 (or more) different antibiotics on a particular day this was counted as 2 (or more) DOT. Cotrimoxazole was analysed when dosed in 960 mg BID, leaving out *Pneumocystis jirovecii*/*Toxoplasma* prophylaxis after allogenic SCT (480 mg QD).

Other secondary outcomes were 30-day mortality, ICU-admission within 30 days after start EAT and blood cultures positive for microorganisms sensitive to imipenem. These outcomes were measured for the neutropenic episodes in which EAT was started. Cases of mortality within 30 days after start of EAT with a carbapenem were reviewed separately by three of the authors (AN, AB and TVDB). Overall mortality, infection-associated mortality and carbapenem preventable mortality were distinguished. Infection-associated mortality was defined as clinical signs and/or microbiologically results compatible with infection at time of death. Carbapenem preventable mortality was defined as infection-associated mortality where continuing EAT possibly could have prevented the adverse outcome, because of a suspected or proven etiologic agent that was carbapenem sensitive.

Positive blood cultures within neutropenia drawn after discontinuation of EAT were analysed, because the goal was to study the possible adverse consequences of early discontinuation of carbapenems (e.g. infection/bacteraemia with carbapenem sensitive microorganisms).

### Data analysis

Analysis was conducted at the level of the neutropenic episodes. Changes in carbapenem use and vancomycin use were analysed by ITS. Effect of intervention was represented by a step change (representing the immediate effect). A segmented linear mixed regression model was used with a random intercept for individual patients to correct for multiple neutropenic episodes per patient. Analysis was not corrected for confounders. The 95% confidence interval was derived through bootstrapping with 20,000 iterations. For all antibiotics included in this study, the overall use of different antibiotics within period I was compared to period II, expressed in DOT/100 neutropenic days. Data-analysis was performed using SAS enterprise Guide 7.1. Statistical analysis was done using R version 3.5.1.

## Results

### Patients and neutropenic episodes

**Fig. 2:**
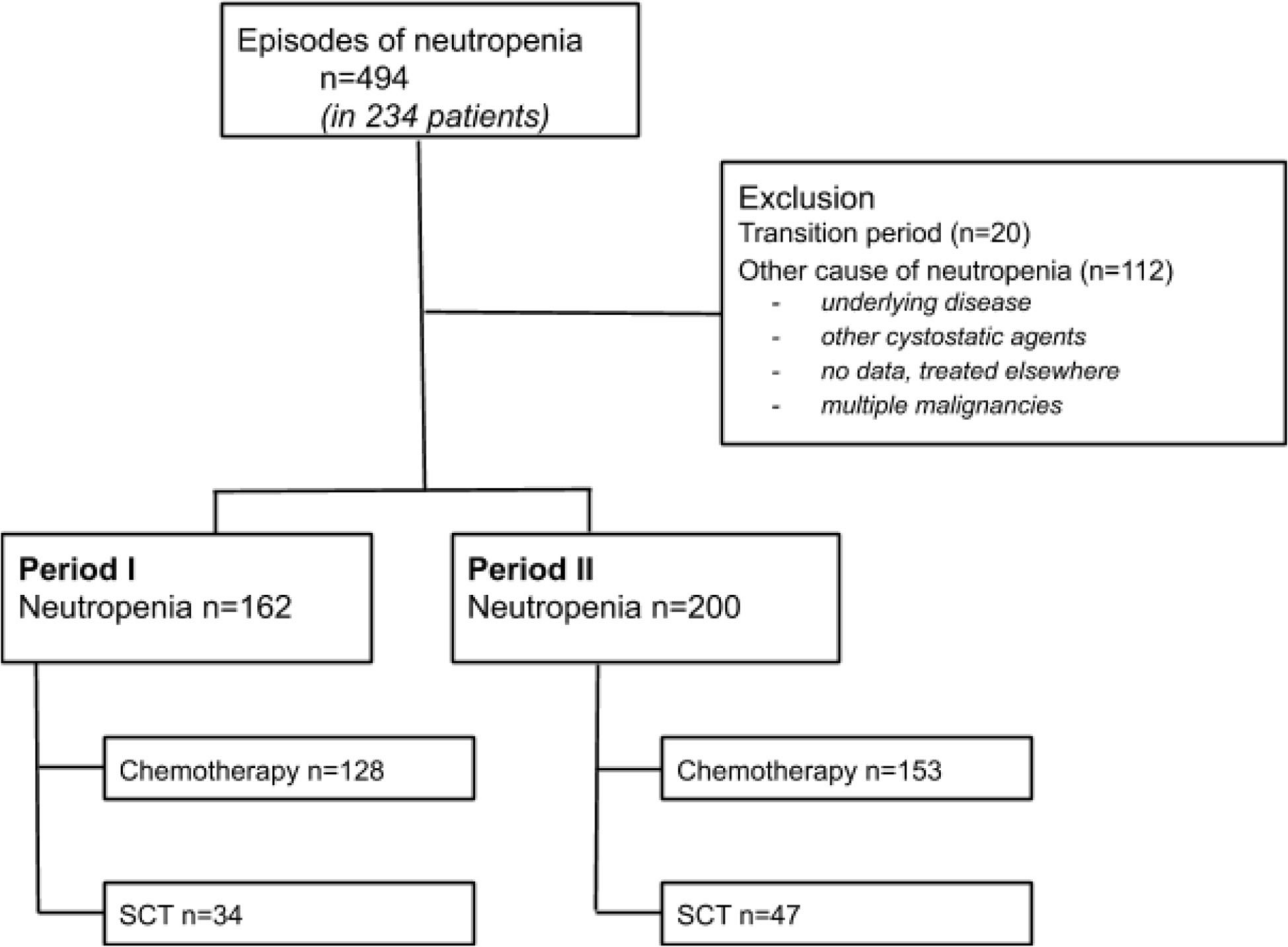
Selection of neutropenic episodes

In total, 234 patients with AML or MDS and neutropenia were identified with a total number of 494 neutropenic episodes. After exclusion of episodes not related to chemotherapy or allogenic SCT and 20 neutropenic episodes during the transition period, 362 neutropenic episodes remained for further analysis (Fig. 2). These neutropenic episodes involved 201 individual patients, of which 184 were diagnosed with AML and 17 with high-risk MDS (Table 1). In AML patients 267 neutropenic episodes were associated with intensive chemotherapy and 72 neutropenic episodes were associated with allogenic SCT (not shown). Further analysis of neutropenic episodes, primary and secondary outcomes, was performed on neutropenic episodes with AML and high-risk MDS patients grouped together. Characteristics of the neutropenic episodes are shown in Table 2. The median duration of neutropenia of all neutropenic episodes combined was 18 days in both period I (IQR 12–25) and period II (IQR 12–26). (Table 2).

**Table 1:** Patient characteristics

		Period I	Period II
**Total**	n	97	104
**Diagnosis**	AML	90 (93%)	94 (90%)
	High-risk MDS	7 (7%)	10 (10%)
**Sex**	Male	46 (47%)	48 (46%)
**Age**	Years ± SD	50.7 ± 14.7	53.4 ± 15.1

*Abbreviations: AML acute myeloid leukaemia; MDS myelodysplastic syndrome*

**Table 2:** Characteristics of neutropenic episodes

	**Neutropenic episodes in period I**					**Neutropenic episodes in period II**				
	Total	Duration in days		Carbapenem started		Total	Duration in days		Carbapenem started	
	*n*	*median (IQR)*		*n*	*%*	*n*	*median (IQR)*		*n*	*%*
Chemotherapy	128	19	(14–26)	105	(82)	153	18	(13–25)	123	(80)
Allogenic SCT	34	11	(8–17)	11	(32)	47	12	(9–26)	29	(62)
**Total**	162	18	(12–25)	116	(72)	200	18	(12–26)	152	(76)

### Primary outcome

**Fig. 3:**
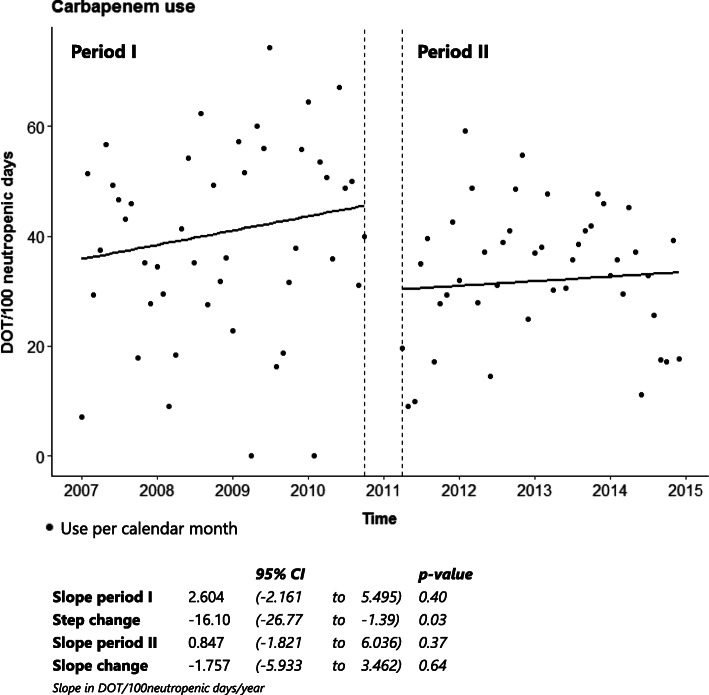
ITS analysis of carbapenem use

**Fig. 4:**
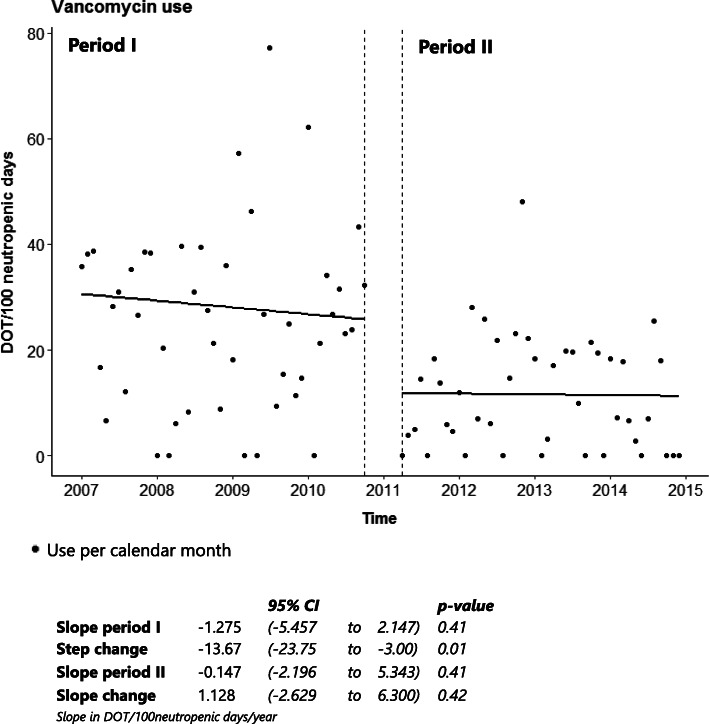
ITS analysis of vancomycin use

ITS analysis showed a significant decrease in carbapenem use with a step change of − 16.1 DOT/100 neutropenic days (95% CI − 26.77 to − 1.39, *p* = 0.03), as shown in Fig. 3. In both period I and II there had been upward trends in carbapenem use, but these were not significant. Overall carbapenem use within period I was 40.3 DOT/100 neutropenic days versus 31.6 DOT/100 neutropenic days in period II, a decrease of 21.6% (Table 3). Vancomycin use decreased significantly after intervention with a step change of − 13.7 DOT/100 neutropenic days (95% CI − 23.75 to − 3.0, *p* = 0.01) (Fig. 4). In both period I and period II there had been a downward trend in vancomycin use, though not significant. Overall vancomycin use decreased from 26.7 DOT/100 neutropenic days in period I to 12.1 DOT/100 neutropenic days in period II, a decrease of 54.7% (Table 3).

**Table 3:** Overall antibiotic use

		Days of therapy/100 neutropenic days	
		Period I	Period II
**Therapeutical agents**	Carbapenems	40.3	31.6
	Vancomycin	26.7	12.1
	Ceftazidime	0.1	0.3
	Ceftriaxone	0.8	0.8
	Piperacillin/tazobactam	0.2	1.1
	Penicillin	3.1	3.8
	Aminoglycosides	0.5	0.6
**Prophylactic agents**	Clindamycin	10.9	7.0
	Ciprofloxacin	65.8	60.1
	Cotrimoxazole *	3.1	5.6
	Cefazolin	4.1	15.0
**Total**		155.6	138.0

*dose > 960 mg BID

### Secondary outcomes

The overall use of several antibiotics in period I and within period II is shown in Table 3. The results show an increase in therapeutically used antibiotics other than vancomycin and carbapenems. However, this increase was small compared to the decrease in vancomycin and carbapenem use. Total use of therapeutic antibiotics decreased from 71.7 DOT/100 neutropenic days in period I to 50.3 DOT/100 neutropenic days in period II. Total use of prophylactic antibiotics in period I (83.9 DOT/100 neutropenic days) was slightly higher in period II (87.7 DOT/100 neutropenic days). Total antibiotic use (therapeutic and prophylactic antibiotics combined) decreased from 155.6 DOT/100 neutropenic days in period I to 138.0 DOT/100 neutropenic days in period II, a reduction of 11.3% (Table 3).

No apparent difference in mortality associated with an infection that could have been prevented with continued carbapenem treatment was identified. The overall mortality within 30 days after starting EAT was 5.6% (in 15/268 neutropenic episodes in which EAT was started). Eleven fatal cases were associated with infection: two out of 116 (1.7%) in period I and nine out of 152 (5.9%) in period II. However, these cases were all classified as unlikely to be preventable with continuation of carbapenem therapy for the following reasons. Firstly, a carbapenem was actually continued (*n* = 5), according to the protocol in hemodynamically instable patients. Secondly, the pathogen was not susceptible to carbapenems (*n* = 3), i.e. mucormycosis, pulmonary aspergillosis and invasive candidiasis with *Candida glabrata.* Thirdly, appropriate targeted therapy was given, based on microbiological findings (*n* = 1), i.e. a case of *Clostridium difficile* pseudomembranous colitis in combination with *Stenotrophomonas maltophilia* bacteraemia, treated with metronidazole and levofloxacin. Finally, presumed appropriate targeted therapy was given based on a clinical diagnosis (*n* = 2), i.e. a patient with cellulitis treated with cefuroxim and a patient with neutropenic enterocolitis treated with a carbapenem which was interrupted for 1 day. Twelve days after interruption of EAT this last patient died of a bleeding in the liver, which was considered not to be related to colitis. For all cases in which EAT was discontinued, ciprofloxacin prophylaxis was administered according to the protocol.

No notable difference between the number of ICU-admissions within 30 days after starting EAT was observed, 9/116 neutropenic episodes in period I (7.8%) and 9/152 neutropenic episodes in period II (5.9%).

During the study period, blood cultures obtained after discontinuation of EAT were positive in 56 of 268 neutropenic episodes (20.9%). In six of these neutropenic episodes blood cultures were positive for microorganisms sensitive to imipenem, i.e. *Streptococcus mitis* (*n* = 1)*, Escherichia coli* (*n* = 1)*, Clostridium perfringens* (*n* = 1), *Pseudomonas aeruginosa* (*n* = 1) and *Enterococcus* spp. (*n* = 2). Four out of 116 (3.4%) of these positive blood cultures were obtained in period I and two out of 152 (1.3%) in period II. One blood culture in period II was positive for *Enterococcus* spp. and one for *Pseudomonas aeruginosa*. In all cases adequate therapy was started when blood culture results became available. No fatal outcomes were recorded within 30 days after early discontinuation of EAT. More details are shown in Table 4.

**Table 4:** Positive blood cultures with carbapenem sensitive microorganisms within 30 days after discontinuation of EAT

	Patient (sex, age, diagnosis)	Duration of initial EAT	Micro-organism in blood culture	Days between discontinuation of EAT and positive blood culture	Focus of infection	Treatment
**Period I**	F 51, AML	11	*Enterococcus* species	4	Central venous catheter	Vancomycin, followed by amoxicillin
	F 44, AML	7	*Streptococcus mitis*	6	Sinusitis	Restart EAT with a carbapenem
	F 65, MDS	7	*Escherichia coli*	11	Urosepsis	Restart EAT with a carbapenem, followed by ceftriaxone
	M 59, AML	16	*Clostridium perfringens*	7	Translocation of infected trombus	Restart EAT with a carbapenem + vancomycin
**Period II**	M 49, AML	4	*Pseudomonas aeruginosa*	6	Dental focus	Piperacillin/tazobactam + tobramycin
	M 42, AML	5	Enterococcus species	17	Unknown	Vancomycin

Abbreviations: *F* female; *M* male; *AML* acute myeloid leukaemia; *MDS* myelodysplastic syndrome; *EAT* empirical antibiotic treatment

## Discussion

This study shows a reduction in carbapenem and vancomycin use after introduction of a protocol promoting early discontinuation of EAT in high-risk haematology patients with febrile neutropenia. According to this protocol, EAT with carbapenems is discontinued after 3 days (with bacterial prophylaxis restarted), regardless of fever, provided blood cultures remain negative, patients are hemodynamically stable and do not have a pulmonary infection with unknown etiology. Also the standard use of vancomycin as EAT is discouraged in the new protocol, following the IDSA guideline [1]. Importantly, both carbapenems and vancomycin are classified by the WHO as key targets of antimicrobial stewardship programs [7].

In period II, carbapenems were started slightly more often compared to period I (76% vs. 72%, Table 2). Despite the increased frequency of starting carbapenems, ITS showed a significant reduction (step change, Fig. 3) and the overall use was decreased by 21.6% (Table 3). In addition to the reduction of EAT with carbapenems, there was a 54.7% reduction of vancomycin use. Besides, the effect of intervention on carbapenem and vancomycin use seemed to be sustained over period II.

Analysis of administration of other therapeutically used antibiotics did not show striking increases, indicating that the restrictive use of carbapenems and vancomycin was not compensated by replacement with other broad-spectrum antibiotics. There was a modest increase in the use of prophylactic antibiotics after introduction of the protocol. This can be attributed to the increased prophylactic use of cefazoline and cotrimoxazole. Nonetheless, total antibiotic use (prophylactic and therapeutically used agents combined) decreased with 11.3%.

The reduction of EAT after protocol implementation did not lead to an increase in adverse events. Although all-cause mortality was higher in period II, detailed analysis did not reveal causality between early discontinuation of EAT and fatal outcomes. Secondly, there was no notable difference in ICU admittance within 30 days after discontinuing EAT between the two periods. Thirdly, in both periods there were some episodes of bacteraemia with a carbapenem-susceptible microorganism within 30 days after stopping EAT. However, all these episodes of bacteraemia were treated adequately and no mortality within 30 days after early discontinuation of EAT was observed in these cases.

Shortening the course of EAT in haematological patients with febrile neutropenia remains a subject of debate [13, 14] while there is increasing evidence supporting a shorter course of EAT in these high-risk haematology patients [8–10]. Many of these studies focus on the safety of the Fourth European Conference on Infections in Leukaemia (ECIL-4) recommendation to discontinue EAT after 48 h of apyrexia [3, 8, 9, 15]. This policy reduces EAT in comparison to previous recommendations [8, 9, 15]. However, broad-spectrum antibiotic use may be reduced even further, because febrile neutropenia is frequently unrelated to infection [4–6]. In this respect, the study of Slobbe et al. is interesting, describing a protocol to discontinue EAT after 3 days, regardless of persisting fever [10]. The authors did not document any mortality related to an untreated bacterial infection and concluded early discontinuation of EAT is safe, although the study lacked a control group. We compared mortality and other measures of safety before and after implementation of a similar protocol, thereby providing a historical control group.

This study has limitations. Most important, the retrospective design of this before-after study makes it impossible to take into account all time-related factors that could have influenced the outcome measures. Therefore, the results can only be interpreted as descriptive. Two potential time-related factors, i.e. changes in patient population and treatment intensity, may influence the outcomes. More vulnerable patients (e.g. more serious comorbidities) have become eligible for haematological treatment regimens and conditioning regimens for allogenic SCT have become more intense. This may have led to more severe morbidity in the second (intervention) cohort. Despite these changes, potentially leading to an overestimation of adverse events in period II, this study shows a decrease in consumption of EAT and no increased infection-associated morbidity and mortality. However, the frequency of the studied adverse events (e.g. mortality, ICU-admission and positive blood cultures with imipenem sensitive microorganisms) is relatively small, so the study may be underpowered for the safety outcomes. No data on temperature was available. However, patients were considered febrile when carbapenems were started because febrile neutropenia was an almost exclusive indication for administering carbapenems.

## Conclusion

In conclusion, this study shows that introducing a protocol advocating early discontinuation of EAT in AML and high-risk MDS patients with febrile neutropenia during chemotherapy or conditioning for allogenic SCT, resulted in decreased use of carbapenems and vancomycin, without apparently compromising patient safety. In addition to current recommendations, early discontinuation of EAT without the explicit need for apyrexia is worth further exploration in stable haematological patients with febrile neutropenia.

## Data Availability

The datasets generated and analysed during the current study are not publicly available due to confidentiality, but are available from the corresponding author on reasonable request for researchers who meet the criteria for access.

## Abbreviations

AML: Acute myeloid leukaemia
BID: Bis in die/2 times a day
DOT: Days of therapy
EAT: Empirical antibiotic treatment
GLIMS: General Laboratory Information Management System
ITS: Interrupted time series
MDS: Myelodysplastic syndrome
QD: Quaque die/once a day
QID: Quater in die/4 times a day
SCT: Stem cell transplantation
TID: Ter in die/3 times a day
UMCU: University Medical Centre Utrecht
UPOD: Utrecht Patient Oriented Database
